# Implementation evaluation of a professional development program for comprehensive school physical activity leaders

**DOI:** 10.1016/j.pmedr.2020.101109

**Published:** 2020-05-05

**Authors:** Russell L. Carson, Ann Pulling Kuhn, Justin B. Moore, Darla M. Castelli, Aaron Beighle, Katie L. Hodgin, Brian Dauenhauer

**Affiliations:** a Louisiana State University, School of Kinesiology, 112 Long Fieldhouse, Baton Rouge, LA 70803, USA; b University of Northern Colorado Active Schools Institute, School of Sport and Exercise Science, Gunter Hall, Box 39, Greeley, CO 80639, USA; c Wake Forest School of Medicine, Department of Family and Community Medicine, Medical Center Boulevard, Winston-Salem, NC 27157, USA; d University of Texas at Austin, Department of Kinesiology and Health Education, 2109 San Jacinto Blvd., Austin, TX 78712, USA; e University of Kentucky, Department of Kinesiology and Health Promotion, Lexington, KY 40506, USA

**Keywords:** Physical Activity Leader, School champion, Whole-of-school, Coordinator, School health

## Abstract

The purpose of this study was to conduct an implementation monitoring evaluation of a yearlong comprehensive school physical activity program (CSPAP) professional development program across eight multi-state physical education (PE) teacher cohorts. Mixed-method data were collected during a three-year implementation period via workshop attendance sheets and evaluations, post-workshop implementation plans and artifacts, and follow-up phone interviews to enumerate and evaluate the program’s process of recruitment, reach, dose delivered, dose received, fidelity, and context. Recruitment strategies reached a total of 234 PE teacher attendees across eight workshops, with 77 PE teachers (primarily female, elementary, public school teachers) completing all program requirements. Facilitators among full program completers were participation incentives and network opportunities, while common inhibitors were difficulty with online technology and perceptions of added workload. Completers submitted implementation plans with at least three action steps, ranging from 4 to 7 months to accomplish, that predominately commenced with securing administration approval as the first step (81%), focused on implementing student physical activity initiatives beyond PE (76%), and evidenced with mostly picture artifacts (78%). Implementation was facilitated by the presence of multilevel support at school and an elevated image of PE and PE teachers at school, and was inhibited by scheduling constraints, unrealistic planning, and conflicting perceptions of physical activity and PE. Overall, this evaluation reveals unique perspectives of PE teachers regarding schoolwide PA promotion and informs future efforts to target and effectively support CSPAP leaders.

## 1 Introduction

Despite the recognized benefits of physical activity (PA), few youth meet national PA recommendations (Centers for Disease Control and Prevention, 2017b, Troiano et al., 2008, U.S. Department of Health and Human Services, 2008). A variety of school-based strategies have been advocated for promoting youth PA (CDC, 2017b), but at the forefront have been multicomponent, whole-of-school approaches (Institute of Medicine [IOM], 2013). One identified as the national framework for increasing the school PA levels of youth is a Comprehensive School Physical Activity Program (CSPAP; Centers for Disease Control and Prevention, 2015, Centers for Disease Control and Prevention, 2017a, Centers for Disease Control and Prevention, 2017b). The goal of a CSPAP is to develop an active school culture conducive to promoting lifelong PA across five integral components: a) physical education (PE), b) PA during school, c) PA before and after school, d) staff involvement and e) family and community engagement (SHAPE America, 2015a).

**Fig. 1:**
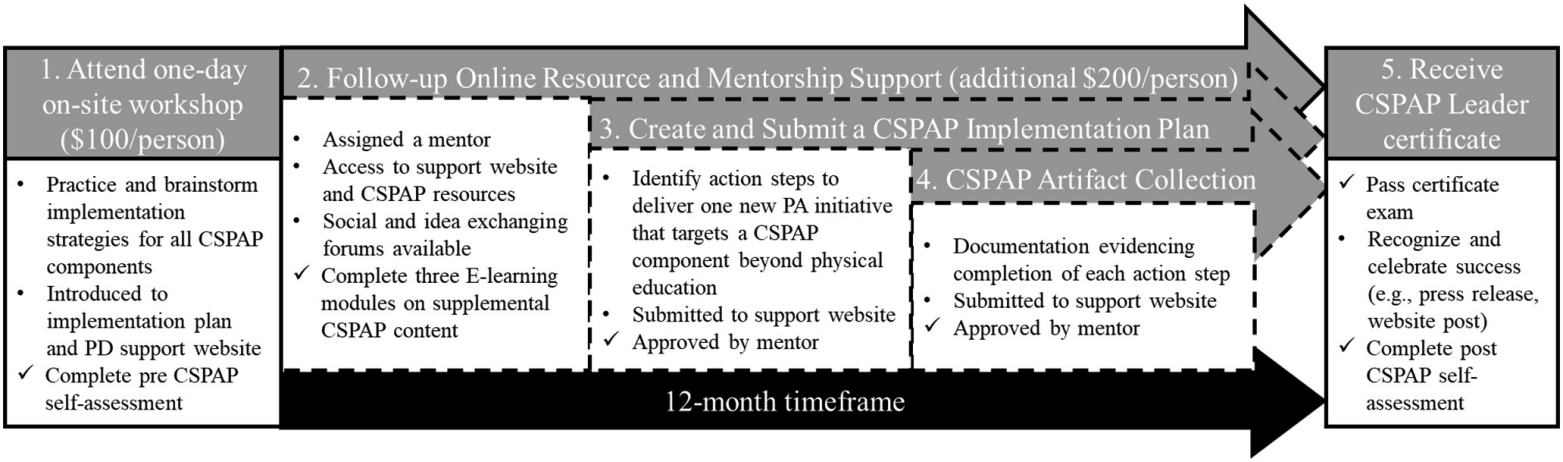

To effectively deliver a CSPAP, schools should have an on-site champion to spearhead such efforts (Carson et al., 2014a, Centers for Disease Control and Prevention, 2013). A national CSPAP professional development program, originally termed the Director of Physical Activity (DPA) certification program (Carson, 2012), now the Physical Activity Leader Learning System (SHAPE America, 2015b),^1^ was established to train school professionals as school leaders of CSPAP implementation. PE teachers are generally the audience for this program because of their professional expertise, access to a majority of students, and available resources to provide a developmentally appropriate PA curriculum (Castelli and Beighle, 2007). However, they often have limited training in the coordination of PA opportunities across the school curriculum and context (Beighle et al., 2009). PE teachers may be interested in learning more about implementing school-wide PA opportunities, earning required professional development credits, or the C-DPA title (Carson, 2012). A six-person task force was commissioned by the Society for Health and Physical Educators (SHAPE) America^2^ and, after a brief pilot phase in the summer prior to year 1, the program was marketed to SHAPE America members and networks nationwide and served as the foundational CSPAP professional development program available for PE teachers (see Carson, 2013). Full completion of the professional development program is a fluid 12-month process that contains a series of criteria overviewed in Fig. 1. Criteria, along with guiding CSPAP duties of trained teachers, have been described in greater detail elsewhere (Carson, 2012).

## 2 Implementation of a CSPAP professional development program

Process evaluation has been identified as important to quantify the fidelity of program implementation, equally as important as evaluating the impacts and outcomes of an intervention (Bartholomew et al., 2006, Glasgow et al., 2004). The information gathered through implementation monitoring can be helpful in determining the acceptability and feasibility of the program and can inform the development of a formal implementation protocol for both formative purposes (i.e., distinguish fine-tuned modifications for quality improvement) and summative purposes (i.e., provide input for the future development of comparable programs; Riley et al., 2010, Saunders et al., 2005). Accordingly, practitioners and researchers may use process data collected through implementation monitoring to understand how the program works in a practical setting, and why the program was, or was not, successfully delivered (Steckler and Linnan, 2002).

Six process aspects should be considered when evaluating health promoting programs: recruitment, reach, dose delivered, dose received, fidelity, and context (Saunders et al., 2005, Steckler and Linnan, 2002). *Recruitment* refers to the procedures used to approach and attract program participants. *Reach* is the participation rate in the program, often measured by attendance rates and characteristics of participants. *Dose delivered* refers to the degree of completeness with which the intended program elements were provided to participants. *Dose received* is the extent to which participants were exposed to, utilized, and/or were satisfied with the intended program elements. *Fidelity* is the extent to which quality program interventions were implemented as planned. *Context* refers the potential barriers and facilitators that could be encountered when implementing program interventions. These six process aspects have served as conceptual guides for monitoring the implementation of school-based PA programs (Hall et al., 2012, McKenzie et al., 1994, Saunders et al., 2006).

The purpose of this study was to conduct a mixed-methods process evaluation of the delivery and implementation of a yearlong CSPAP professional development program across eight multi-state PE teacher cohorts over a three-year implementation period. Quantitative and qualitative data were collected from participating PE teachers to describe the *recruitment*, *reach*, *dose delivered*, *dose received*, *fidelity*, and *context* of the implementation to inform the optimal design, dissemination, and implementation of CSPAP professional development programs.

## 3 Methods

### 3.1 Study population

A CSPAP professional development program was implemented in three consecutive delivery periods (one per calendar year; year 1, 2 and 3). During this three-year timeframe, there were a total of 440 participants in the professional development program from 24 states in the U.S. and one Canadian province. Participants were PE teachers (84.8%), higher education faculty from PE teacher education programs (10.0%), health or PE district staff (3.6%), and PE graduate students (1.6%; Carson, 2013). The PE teacher participants (*N* = 373) were the target population for this evaluation with the exception of 47 PE teachers who were part of the Year 1 planning and pilot phase, and the 92 PE teachers who participated in the workshop only (see Centeio et al., 2014). Therefore, the study sample included only the PE teachers participating in aspects of the 12-month follow-up period of the professional development program (*n* = 234).

### 3.2 Design overview

Similar to previous process evaluations of school PA promotion interventions (de Meij et al., 2013), a convergent mixed-method design was applied in this study. Quantitative and qualitative assessments of the six process aspects were combined to evaluate the delivery and implementation of the CSPAP professional development program (Creswell and Plano Clark, 2011; see Table 1). Effectiveness data from the project, including self-assessments of school PA offerings and student accelerometer data, have been presented elsewhere (Carson et al., 2014b).

**Table 1:** Design of the Implementation Monitoring Strategy.

Process aspect	Description of implementation monitoring strategy	Data source	Method	Analytic procedure
Recruitment	Procedures used to approach, attract and register participants in the CSPAP PD program	Instructor records	Quantitative Qualitative	Descriptive statistics N/A
	Facilitators and inhibitors participants expressed to attend the onsite workshop.	Interview transcripts	Qualitative	Inductive analysis
Reach	Participation rates and characteristics of teachers who attended the onsite workshop	Workshop attendance sheets	Quantitative	Descriptive statistics
Dose delivered	Number of teachers in training cohorts who were provided the complete PD program as planned (i.e., post-workshop during the 12-month PD timeframe)	Task force records	Quantitative	Descriptive statistics
Dose received	Documented degree of satisfaction with workshop	Workshop evaluations	Quantitative Qualitative	Descriptive statistics Inductive analysis
	The proportion and characteristics of teachers who completed the post-workshop criteria (implementation plan, artifact, certification exam), as denoted in Fig. 1a	Approved implementation plans, Submitted artifacts, Certification lists	Quantitative	Descriptive statistics
	Facilitators and inhibitors teachers encountered from completing these criteria.	Interview transcripts	Qualitative	Inductive analysis
Fidelity	Extent and quality of PA initiatives proposed and implemented in schools.	Approved implementation plans	Quantitative	Descriptive statistics
		Submitted artifacts	Qualitative	Document analysis
Context	Perceived factors (i.e., professional, community, social/political) that facilitated or inhibited the implementation of the new PA initiative in schools from the CSPAP PD program	Interview transcripts	Qualitative	Inductive analysis

*Notes*. Descriptive statistics included cumulative frequencies, percentages, and means. CSPAP = comprehensive school physical activity program; PD = professional development; N/A = not applicable, limited instructor reports to be analyzed qualitatively.

aCSPAP self-assessments have been contained in outcome evaluations reported elsewhere (Carson et al., 2014b)

### 3.3 Process data sources and procedures

Multiple sources of data were accessed throughout the three-year implementation period to evaluate the six implementation components (indicated in parentheses after each data source below). Informed consent was obtained from participants prior to data collection. Study approval was granted by SHAPE America as the managing organization of the professional development program and the Institutional Review Board of the participating universities. All data available to the research team were included in this study.

#### 3.3.1 Task force records (recruitment, dose delivered)

The SHAPE America task force charged to develop the professional development program and its content served in many other capacities including: (a) the program’s principal consultants, (b) mentors during the 12-month professional development timeframe (per period), (c) website support technicians, (c) program evaluators and researchers, and (d) master instructors of the workshops. To fulfill these responsibilities, the task force members maintained regular communication with each other and SHAPE America throughout the three-year implementation period. Modes of communication were five in-person Year 1 meetings during planning and pilot phase, seven conference calls to discuss professional development program evaluation/expansion or research data management (i.e., three in Year 1, three in Year 2, one in Year 3), and monthly e-mail correspondence among the task force co-chairs. Notes taken from these communications, including formal and informal observations, were distributed to the task force co-chairs who also spearheaded this evaluation research.

#### 3.3.2 Workshop attendance sheets (reach)

Teachers interested in attending the one-day on-site workshop provided basic contact information including name, school name and address, e-mail address, and phone number. This information was collected twice by different means: (a) registration data – interested teachers signed up for a training workshop via SHAPE America’s professional development webpage, or by e-mail or phone directly, and (b) attendance data – when teachers arrived at the training workshop via a sign-in sheet. The workshop was free for attendees ($100 value supported by grant funds), and as an additional incentive, attendance sheets were utilized to distribute continuing education credits to workshop attendees.

#### 3.3.3 Workshop evaluations (dose received)

PE teachers who attended the six-hour workshop were asked to complete a 12-question evaluation form at the conclusion of the workshop. Nine questions used 5-point Likert-type scales (e.g., 1 = *strongly disagree* to 5 = *strongly agree*) to quantitatively measure workshop attendees’ ratings of the workshop’s: (a) content and delivery (e.g., “The content was ____ to my job”), (b) the instructor’s characteristics (e.g., “The instructor was knowledgeable”), and (c) their general experience (e.g., “I would recommend this workshop to colleagues”). Three open-ended questions were used to supplement these workshop attendees’ ratings with qualitative data pertaining to the workshop content (“What would you like more information on?”), their general experience (“What was the most impactful part of the workshop”), and their next steps (“What action will you take at your school as a result of this workshop?”). The evaluation form was created by the task force as an expectation of: a) SHAPE America endorsed trainings, and b) earning continuing education credits.

#### 3.3.4 Approved implementation plans (dose received, fidelity)

One main completion criterion during the 12-month professional development timeframe was for PE teachers to develop a plan to implement a new and achievable PA initiative pertaining to a CSPAP component that complements physical education (e.g., to provide PA during school, PE teacher organizes equipment bags for a weekly PA morning club for students). The implementation plan form was introduced to PE teachers near the end of the workshop and teachers were provided time to brainstorm and conceptualize some possible ideas amongst workshop attendees and the instructor. The implementation plan form provided space for PE teachers to identify the overall goal for the new PA initiative, the CSPAP component(s) the initiative would address, and the planned sequence of strategic action steps to accomplish it. Further, PE teachers were asked to outline four elements for each action step on the form: a) necessary resources, b) necessary personnel assistance, c) completion date, and d) an artifact (see next section for description) to document completion. PE teachers furnished an electronic version of their finished implementation plan to their assigned mentor (i.e., mentor was the workshop master instructor for each site) for the 12-month professional development timeframe for review, feedback, and approval. Implementation plan submissions and teacher-mentor transactions occurred primarily through the support website that accompanied the professional development program or individual e-mail. An anonymous sample of approved implementation plans were displayed on the support website as guided examples.

#### 3.3.5 Submitted artifacts (dose received, fidelity)

Another main completion criterion during the 12-month professional development timeframe was the submission of at least one artifact corresponding to each action step on the approved implementation plan. Artifacts were to be representative electronic documents, such as school schedules, revised curriculum, event materials, sample videos, and participant vignettes that supply evidence the action step was completed. The submission, review, and approval process of the artifacts, along with the posting of approved samples on the support website, was comparable to that of the implementation plans.

#### 3.3.6 Certification lists (dose received)

PE teachers were awarded a certificate after completing all criteria in the professional development program (denoted as check marks in Fig. 1). The 12-month follow-up period leading to certification was a $200 charge. The final step included passing a certification exam with a score of 80% or higher. PE teachers were allowed to retake the exam once if they failed their first attempt. At the conclusion of the 12-month timeframe, mentors supplied SHAPE America with the final list of PE teachers who were full completers of the professional development program and, therefore, earned their certification. Becoming certified signified a qualification in best practice knowledge of the current national framework in schoolwide physical activity, endorsed by the leading professional organization of PE teachers (i.e., SHAPE America) and became a prerequisite for related national awards.

#### 3.3.7 Interview transcripts (recruitment, dose received, context)

All PE teachers who attended an on-site workshop and expressed interest in seeking their completion certificate were invited to participate in one in-depth phone interview. The 20 consented PE teachers were categorized into two self-selected groups, matched by sex (7 = female), tenure level (9 = 5 + years of experience) and school locale (7 = urban setting), that were based on completion rates of the professional development program: 10 *full completers* – PE teachers who fulfilled all criteria during the 12-month professional development timeframe, and thereby earned a certificate (see Fig. 1); and 10 *partial completers* – teachers who attended the six-hour workshop, but opted out of some of the post-workshop criteria. Trained interviewers conducted the interviews with each PE teacher individually at the one-year mark after they attended the workshop (also when the full completers received their certificate). Interviews were digitally recorded using computer software and a handheld device for back-up, scheduled on a school day and convenient time for the PE teacher, and lasted an average of 58 min (*SD* = 11 min).

The same interview guide and clarification probes were employed with both teacher groups and corresponded to two sets of questions on the topics of perceived (a) sustainability factors and (b) multilevel benefits of the professional development program and CSPAP implementation. PE teachers were asked four questions related to their perceived sustainability of the professional development program and CSPAP implementation, with specific reference to facilitators and inhibitors of the on-site workshop and the implementation of the new PA initiative outlined on the implementation plan. Two sample questions were: “What has/would have inhibited your participation in the workshop/implementation of your plan?” and “What strategies or approaches have you considered or employed to overcome these inhibitors? The two multilevel benefits questions were: “What impact has your CSPAP implementation has on your – yourself, students, school community?” and “What specific strategies did/could you use to achieve the described benefits from CSPAP implementation?” After the 20 interviews were conducted, audio files were transcribed verbatim by a professional typist and returned to the PE teacher for accuracy checks. Partial completers were paid $50 for their participation, and full completers were paid $150 to help offset the $200 fee associated with completing the remaining criteria during the 12-month professional development timeframe (see Fig. 1).

### 3.4 Data analysis

Quantitative data were analyzed with descriptive statistics consisting of cumulative frequencies, percentages, and means using IBM SPSS Statistics for Windows, Version 24.0 (IBM Corp., Armonk, NY). All data sources, except the workshop evaluations and interview transcripts, contained quantitative data.

Qualitative data were analyzed inductively using two naturalistic methods (Patton, 2015). First, document analysis (Bowen, 2009) was performed on the submitted artifacts to derive insight on their content, meaning and contribution to providing supplemental, but substantive, evidence for the sequential completion of each implementation plan. Open and axial coding procedures (Corbin and Strauss, 2008) were conducted by a researcher, with consultation from an experienced peer debriefer, to organize the predefined codes into emergent categories central to the process evaluation aspect being considered. Second, constant comparative method (Lincoln and Guba, 1985), using the three-step procedures of open, axial and selective coding (Corbin and Strauss, 2008), were performed on the open-ended workshop evaluation questions and interview transcripts both within and across the teacher groups. Two researchers independently read and re-read the transcripts and delineated, via open-coding, standalone blocks of raw data. The researchers then met, accompanied by an experienced peer debriefer, to collectively collapse related and unrelated individual units of data around axes of mutually exclusive categories and subcategories. The same three-person team met again to selectively integrate and refine, in light of disconfirming evidence, the higher-order categories into thematic schemes and subthemes generated by consensus. The validation strategies applied throughout both methods of qualitative analyses were seeking corroboration among different data sources, including quantitative, utilizing an experienced peer debriefer, eliminating outliers through negative case analysis, and double-checking findings with the participants and an external auditor (Creswell and Plano Clark, 2011).

## 4 Results

The findings from the quantitative and qualitative data were compared, related, and integrated to produce main results across the six process aspects that are described below.

### 4.1 Recruitment

Procedures used to approach, attract and register participants in the professional development program occurred at both the national and state level. At the national level, the primary recruitment strategy was the website of SHAPE America, the primary professional organization for health and physical education teachers in the U.S. The CSPAP professional development program, along with others available to the 15,000+ membership community, was posted under professional development offerings where members can register for a reduced rate.

Four recruitment strategies were utilized at the state level. First, school health division coordinators from statewide organizations (e.g., health foundation, Department of Education) in the states where workshops were conducted were contacted and asked to distribute information about the professional development program to PE teachers. Second, Health/PE district coordinators in the states where workshops were conducted were asked to distribute invitations to their PE teachers, along with two follow-up reminders. Third, a recruitment booth was set up at the two-day annual meeting for PE teachers in the home state of the lead researchers. Fourth, PE teacher education faculty from the largest undergraduate PE programs in the home state of the lead researchers were asked to send invitations to recent graduates now employed as PE teachers. PE teachers’ registration data for the workshops are presented in Table 2.

**Table 2:** PE Teacher Participants across Criteria of the CSPAP Professional Development Program.

	Registered for workshop		Attended workshop				Submitted implementation plans		Submitted artifacts		Pass certification exam				
Training site	*n*	Female	*n*	Female	Public schools	Elem schools	*n*	%^a^	*n*	%^a^	*n*	%^a^	Female	Public schools	Elem schools
A. Kansas	38	27	37	27	23	21	23	62%	23	62%	22	59%	16	22	17
B. Kansas	30	19	28	18	21	13	25	89%	22	79%	16	57%	13	16	10
C. Kentucky	26	13	26	15	19	14	12	60%	11	55%	11	55%	6	11	5
D. Massachusetts	31	24	21	17	12	3	6	29%	5	24%	4	19%	2	4	2
E. Massachusetts	25	16	24	15	15	7	5	21%	4	17%	4	17%	3	3	4
F. Louisiana	37	23	37	23	34	17	12	32%	12	32%	12	32%	10	11	7
G. Louisiana	28	20	28	20	21	9	6	21%	6	21%	5	18%	4	4	4
H. Louisiana	33	20	33	20	33	20	8	24%	8	24%	3	9%	3	3	3

Totals (Mean %)	248	162 (68%)	234	155 (66%)	178 (76%)	104 (44%)	97	42%	91	35%	77	33%	57 (74%)	74 (96%)	52 (68%)
Criteria completion %			94%^b^				42%^c^		94%^c^		85%^c^				

*Notes*. Percentages calculated from those attended workshop.

A: Wichita, B: Overland Park, C: Louisville, D. Boston1, E: Boston2, F. Baton Rouge1 G. Baton Rouge2 H. Lafayette

a Percentage of workshop attending teachers who completed this criteria.

b Percentage of registered teachers who attended the workshop.

c Percentage of workshop attending teachers who completed this criterion.

In addition, recruitment was measured through the assessment of teacher-reported facilitators of and inhibitors to attending the on-site workshop (see Table 3). Two facilitation themes emerged from the interviews relative to workshop attendance. Workshop attendees noted that the *availability of funds* to attend the training and the ability to *obtain continuing education credits* facilitated their attendance at the workshop.

**Table 3:** Facilitators and Inhibitors from Interviewed PE Teacher Participants (N = 20) in the CSPAP Professional Development Program across Process and Implementation Monitoring Strategies.

Facilitators	Inhibitors	Subthemes (**bold**) and Representative illustrations
**Recruitment**: Workshop attendance		
Availability of funds (*n* = 13)		“The one thing that I saw that really perked my attention was when I think they [district] offered a scholarship for people to get it [workshop] paid for.” (FC6)
Obtain continuing education credits (*n* = 6)		“That’s [credit] also another hook because of always having to stay up on your professional development.” (FC7)
Network opportunities at workshop (*n* = 2)^a^		And the more professional development I can attend, the more different…people I talk to, other physical education teachers….The more I interact with other people, other professionals, the better I become.” (FC4)

**Dose received**: Completion of post-workshop criteria		
Online modules easy to use (*n* = 5)^a^		“I’m fairly good on the computer, so I didn’t feel that I needed a lot of support with that, and it was very similar to other certification processes that I had gone through. So, I felt like it was pretty self-explanatory.” (FC5)
Build collaborations post-workshop (*n* = 3)^a^		“We worked as a group so I was able to work with two other PE teachers in (City) Public Schools and we were able to collaborate on what we were considering to be evidence … and how you would make it feasible.” (FC5)
	Challenges with technology (*n* = 14)	“So to sit down and fight with the technology for 20, 30 min of a planning time or in the evenings was very frustrating to me.” (FC8)
	Added workload (*n* = 11)^b^	“You have to write out a lesson plan, an implementation plan, actually…It’s just like more meetings or more paperwork. I just—I wasn’t too interested in that.” (PC6)
	Immediate mentorship (*n* = 7)	“I think that this all could have been streamlined a little bit, had I had somebody contact me right after the [workshop]. I felt really lost for little bit of time after that whole day of training.” (FC2)

**Context:** Implementation of PA initiatives in schools		
Presence of multilevel support structure (*n* = 125)		**Important others**: “Permission” granted from *administration* necessary for program initiation (FC1) **Helpful others**: *Classroom teachers* and *parents* when “did the majority of the work” allowed CSPAP leaders time to plan and schedule activities (FC8); building *student* ownership helped reduce teacher workload (FC5)
Elevated image of PE and PE teacher (*n* = 23)		**From self**: “I could be a lazy PE teacher and say “Throw the balls out and go”….But….I can’t do that. We get a bad rap already as it is. So I try to do as much as I possibly can to show the classroom teacher…I’m just as smart as you are.” (FC10) **From others**: “[Classroom teachers] see me in a different light….they seek me out now and ask for ways to spice it up in their classrooms, connect things to movement and we [PE teachers] have a different relationship with them [classroom teachers].” (PC7) **From administration**: “I have never seen anybody going around—she’s [*principal*] like the proud peacock. Which is fine with us, because we are proud that she’s supporting physical activity, physical education….Now she’s supporting more activity in the classroom.” (PC3) **From students**: “I think that the implementation had a huge impact on their [*students*’] actions. I’m seeing them now, advocating and encouraging each other to make healthier choices and to do healthier things. I always find it entertaining when I sort of hear them parrot me.” (FC5)
	Perceived schedule constraints (*n* = 57)	**From multiple roles in schools**^a^: “I’m the athletic director here at our school, plus I teach…health education, physical education, consumerism, and career studies. And I see the kids once a week and I also teach computers so it’s really hard to get everything in.” (FC3) **From rigid, academically prioritized schedules**^b^: “I think that’s one of the biggest frustrations. If you know you have all these great ideas and things you can do, but then realize, ‘I just don’t have the class time to get it done.” (PC1)
	Contextually unrealistic program planning (*n* = 30)	From challenges with weather, gym space, limited equipment, and transportation: “…well, one of the things that inhibits student participation is, they must find their own *transportation* from school at that time.” (FC1)
	Conflicting perceptions of PA and PE (*n* = 23)	**PA is not PE’s responsibility**: I think…the whole concept of physical activity is very new. Some Phys Ed teachers don’t feel that the physical activity is their responsibility. They are Phys Ed teachers, not physical activity leaders.” (PC10) “I don’t think enough PE teachers see themselves as the professional they need to be.” (FC7) **PA is the beginning of the end for PE**: I was trying to call people and…get them on board but too many PE teachers felt threatened by the word “structured” recess….They felt like, well this is one step in the direction of them taking away PE and saying they [students] just get recess and it will be structured and led by a recess aid.” (PC7)

*Notes.* Themes and subthemes listed in order of prominence. FC = full completers: earned certification by fulfilling all criteria throughout 12-month timeframe; PC = partial completers: attended on-site workshop, but opted out of some post-workshop criteria; PE = physical education; PA = physical activity.

a Among full completers only.

b Among partial completers only.

### 4.2 Reach

As presented in Table 2, out of the 248 registered, a total of 234 PE teachers (94%) attended one of eight workshops (i.e., cohorts) offered in one of four states (KS, KY, MA, LA). The majority of workshop attendees identified as female (68%) from secondary (56%) and public schools (76%). Attendee characteristics were dissimilar to the percentage distributions of the national teacher population. According to the National Center for Education Statistics (2018), PE teachers are mostly male (60.8%), and the majority of teachers teach in elementary (51%) and public (80%) schools.

### 4.3 Dose delivered

A total of 77 PE teachers across eight cohorts (*M* = 10 teachers/cohort; Range 3–22 teachers/cohort) were delivered the professional development program as planned by fully completing the criteria across the 12-month follow-up period and earning a certification.

### 4.4 Dose received

Workshop evaluation scores are presented in Table 4, Table 5. Workshops were consistently rated by PE teachers very favorably with an average question rating of 4.74 out of 5.00 (*SD* = 0.14; Range 4.58–4.89). Feedback was regularly reviewed and addressed after each workshop.

**Table 4:** Average Workshop Evaluation Scores by Questions across Cohorts.

	Cohort						
Question item	A. Kansas (*n* = 37)	B. Kansas (*n* = 28)	F. Louisiana (*n* = 37)	G. Louisiana (*n* = 28)	H. Louisiana (*n* = 33)	All (*N* = 163)	
						*M*	*SD*
1. The workshop achieved its objectives.			4.69	4.67	4.94	4.77	0.15
2. The workshop met my expectations.			4.41	4.56	4.76	4.58	0.18
3. The information presented was:			4.62	4.39	4.76	4.59	0.19
4. The content of the workshop was……to my job.			4.72	4.78	4.52	4.67	0.14
5. The instructor was knowledgeable.			4.83	4.89	4.94	4.89	0.06
6. The instructor was well-prepared and organized.			4.86	4.83	4.97	4.89	0.07
7. The instructor was engaging.			4.86	4.83	4.94	4.88	0.05
8. What word best describes your overall experience?			4.66	4.61	4.73	4.67	0.06
9. I would recommend this workshop to colleagues.			4.62	4.89	4.79	4.77	0.14
^a^How likely are you to recommend a colleague?	4.95	4.91					

Overall *M*	4.95	4.91	4.70	4.72	4.82	4.74	
Overall *SD*	–	–	0.14	0.17	0.15	0.12	
Response Rate	26 (70%)	17 (61%)	29 (78%)	18 (64%)	33 (100%)	123 (75%)	

*Notes*. Table includes workshop evaluation data available to research team. Five-point Likert scale used (1 = strongly disagree, 3 = neutral opinion, 5 = strongly agree). Table reflects collected or available data.

^a^End-of-workshop evaluation question asked in cohorts A and B only (i.e., Q1-Q9 not asked).

**Table 5:** Open-ended Responses to Participation Evaluation Form (N = 134).

Question	*n*	Representative illustrations
*1. What was the most impactful part of the workshop? (N = 88)*		
Getting new ideas	42	“Lots of good ideas; loved knowing about the other sources for energizers”
In-depth discussions about CSPAP objectives	19	“The segment discussing CSPAP objectives”
Hands on participation in learning of activities for students	16	“Actually being able to participate in what we were being lectured on; activities”
Opportunity to network with colleagues	7	“Getting in a room full of people who have similar interests/loves/wants. Everyone is excited about physical activity – How great is that? Many fresh ideas!”

*2. What action will you take at your school as a result of this workshop? (N = 89)*		
Implement PA programs	68	“Trying to implement a couple simple fitness activities that may grow into a larger school role.”
Meet with administration to discuss PA programs	11	“Share what I’ve learned with the administrators at this workshop.”
Present CSPAP information to faculty and parents	10	“Bring information to school and present this same information to other teachers.”

*3. What would you like more information on? (N = 47)*		
More classroom activities	25	“Impact of different activities
The online certification process	10	“The actual online certification process”
How to get admin. or local universities on board	6	“The certification impact for schools; what to tell admin to get them on board”
Getting communities involved	4	“How to get community involvement”
Getting low socio-economic schools involved	2	“Working with inner city schools and the needs/demands”

*4. Other suggestions, comments, or recommendations. (N = 35)*		
Very informative, engaging, and motivating workshop	25	“This was an excellent training. You have motivated me and taught me so much.”
Make trainings available more often	3	“Awesome presentation and fulfilling. Make this training available more often.”
Separate trainings for elementary/middle/high school	2	“One training for middle/high school and one for elementary school”

*Notes*. Response categories listed in order of prominence by question. *N* = individual units of data.

The main post workshop criteria depicted with checkmarks in Fig. 1 were that teachers develop and upload to a website: (a) an approved implementation plan outlining the multiple steps needed to implement the new CSPAP initiative, (b) at least one representative artifact (i.e., program evidence such as pictures) per action step approved by an assigned mentor, and (c) pass (80% or higher) a certification exam. The proportion and characteristics of PE teachers completing these criteria across the 12-month follow-up timeframe of the CSPAP professional development program are presented in Table 2.

Ninety-seven PE teachers (42% of total workshop attendees) submitted an approved implementation plan. Among these 97 teachers with approved implementation plans, 91 (94%) submitted approved artifacts, and 77 (85%) completed the 12-month professional development program and earned certification. The PE teachers who completed the program and earned certification were largely female (74%) in elementary (68%) public schools (96%), more closely representing the preponderant distribution of the national teacher population (NCES, 2018).

Dose received was also measured by the reported facilitators and inhibitors teachers encountered from completing these criteria (see Table 3). Two facilitators and three inhibitors emerged as themes for completing post-workshop criteria. Teachers noted that the online certification process was easy to use and that the opportunity to network facilitated completion of the criteria. Additionally, teachers perceived difficulty with technology as an inhibitor to the completion process, along with a perception of added workload and mentorship delays by the trainers.

### 4.5 Fidelity

To assess the extent of the PA initiatives proposed for implementation in participating teachers’ schools, non-PE focused implementation plans (summarized in Table 6) were evaluated for the CSPAP component being targeted, how many steps teachers thought were needed to implement the initiative, and how long (in months) and what resources and supports were deemed as essential for implementation. This information was self-reported by the PE teachers during the planning stages prior to implementing the initiative at their school. The CSPAP component targeted in the implementation plan varied considerably, with PA during school initiatives as most commonly proposed (49%) followed closest by before/after school PA (27%) initiatives. Nearly all teachers (99%) identified a minimum of three action steps, ranging from 4 to 7 months in duration to complete, necessary to implement the new PA initiative at their school. As indicated in Table 7, 11 different categories of tasks were included in the implementation plans with the most common tasks being inform faculty (88%) and implementation (83%). The predominant tasks were identified by action step (bolded in Table 7).

**Table 6:** Summary of Submitted Implementation Plans by CSPAP Component and Most Frequent Physical Activity (PA) Initiatives within CSPAP Component (Italics) (N = 85).

CSPAP component	Frequency	*M* Steps	Resources	Support	Time (months)
*PA Initiatives*					
**During school**	**42**	5	Faculty and staff (*n* = 21) Paper materials (*n* = 16) Technology (n = 13)	Faculty and staff (*n* = 94) Students (*n* = 12) Parent/community volunteers (*n* = 7)	4
*Brain breaks*	21				
*Walking/running*	6				
*Activity awards*	4				

**Before/after school**	**23**	5	Paper materials (*n* = 24) Technology (*n* = 16) Facilities (*n* = 14)	Faculty and staff (*n* = 63) Parent/community volunteers (*n* = 11) Students (*n* = 9)	5
*Morning PA program*	7				
*Walking program*	6				

**Family/community Engagement**	**7**	5	Paper materials (*n* = 10) Faculty and staff (*n* = 4) Parent/community volunteers (*n* = 4)	Faculty and staff (n = 28) Parent/community Volunteers (*n* = 8) PTA (*n* = 2)	7
*Family night*	5				

**Multiple components**	**7**	5	Faculty and staff (*n* = 10) Technology (*n* = 6) Printed materials (*n* = 4)	Faculty and staff (*n* = 19) PTA (*n* = 2) Parent/community volunteers (*n* = 1)	6
*Faculty, students*	4				
*Faculty, students, & parents*	3				

**Staff involvement**	**6**	5	Technology (*n* = 6) Equipment (*n* = 3) Time (*n* = 3)	Faculty and staff (*n* = 14) Community sponsors (*n* = 2)	6
*Wellness program*	3				
*Fitness room*	2				
**Total**	**85**				

*Notes*. Table excludes submitted implementation plans focused on the CSPAP component of physical education (*n* = 12). Teacher-identified resources and support needed for implementation appeared multiple action steps: Faculty and staff (e.g. administration, classroom teachers, office staff). Paper materials (e.g. construction paper, surveys, flyers). Technology (e.g. computers, emails, PowerPoints). Facilities (e.g. classrooms, gym, playground). Equipment (e.g. balls, jump ropes, cones). Time (e.g. teacher in-service, afternoon meeting). Volunteers (e.g. parents, community members).

**Table 7:** Teacher-Identified Goal by Action Step (N = 85 Teachers).

	*n*	Step 1		Step 2		Step 3		Step 4		Step 5		Step 6	
		*n*	%	*n*	%	*n*	%	*n*	%	*n*	%	*n*	%
1. Administration approval	43	**35**	81	7	17	1	2	0	0	0	0	0	0
2. Inform faculty	75	21	28	**24**	32	17	23	10	13	3	4	0	0
3. Prepare materials/venue	66	2	3	16	24	**24**	36	15	23	8	12	1	2
4. Implementation	71	2	3	4	6	10	14	**20**	28	**32**	45	**3**	4
5. Event/initiative planning	57	15	26	16	28	15	26	7	13	4	7	0	0
6. Advertisement	29	3	10	9	31	9	31	7	24	1	4	0	0
7. Seek assistance/supervision	24	3	13	7	29	5	21	7	29	2	8	0	0
8. Evaluation	23	1	4	0	0	2	9	3	13	16	70	1	4
9. Inform parents/approval	3	0	0	1	0.3	1	0.3	1	0.3	0	0	0	0
10. Modify initiative	3	0	0	0	0	0	0	1	0.3	1	0.3	1	0.3
11. Generate knowledge about initiative	2	2	100	0	0	0	0	0	0	0	0	0	0

Totals		85 (100%)		84 (99%)		84 (99%)		70 (82%)		67 (64%)		6 (9%)	

*Notes.* Table excludes submitted implementation plans focused on the CSPAP component of physical education (*n* = 12). Numbers represent how many teacher reported a specific objective overall and by action step. Bold font represents the most frequent goal per action step.

The quality of the targeted CSPAP components and PA initiatives implemented in schools was assessed via qualitative document analysis (Bowen, 2009) of the 360 artifacts submitted by PE teachers. As presented in Table 8, qualitative forms of artifacts were submitted to evidence action steps 56% of the time, while quantitative forms of artifacts were submitted 44% of the time. Qualitative artifact types were largely pictures (78%), while quantitative artifact types were more diverse with sign-in sheets (15%), PA initiative write-up (14%), PA initiative calendar (13%) and administrative approval letter (10%) as the most frequent quantitative form of artifact submitted by PE teachers. Overall, the quality of the artifact data mainly represented a selective, uneven, firsthand viewpoint (Bowen, 2009). That is, artifacts, particularly the photos, documented details on some aspect of the initiative from mostly the perspective of the teachers, rather than a comprehensive, balanced snapshot of the initiative from varying viewpoints.

**Table 8:** Types of Submitted Artifacts (N = 360).

Quantitative	n	%	Qualitative	n	%
Sign-in sheet	32	15	Picture	168	78
Initiative summary/write-up	31	14	Reward board	13	6
Calendar/handout	28	13	Newsletter	7	3
Admin approval email/letter	22	10	Flyer	7	3
Teacher meeting agenda	12	6	Parent feedback	6	2
Teacher email correspondence	12	6			
Participant consent form	11	5			
Presentation notes	11	5			
Total	159	44	Total	201	56

### 4.6 Context

The perceived professional, community, and social/political contextual factors that facilitated or inhibited the implementation of the new PA initiative in schools from the CSPAP professional development program were revealed from the interview data using inductive analysis both within and between full completers and partial completers groups (Patton, 2015). A total of 533 individual units of data (310 full completers, 223 partial completers) were identified from the initial open coding that included but were not limited to the following: academic pressure, transportation, funding, supportive administration, traditional thinking, lack of equipment, and quality PE program. The researchers met and collapsed the individual units of data for each group into a total of 101 subcategories (e.g. 29 full completer facilitators, 22 full completer inhibitors, 30 partial completer facilitators, and 20 partial completer inhibitors), and solidified 21 major categories (e.g., student leadership, resources for implementation, poor planning, and reluctant classroom teachers) across groups (10 full completers, 11 partial completers). Guided by the constant comparison method (Lincoln and Guba, 1985), five major themes emerged from the final categories along with four subthemes by group. The researchers agreed these procedures saturated the data and final themes reflected the interview data.

Two facilitator themes (*presence of a multilevel support structure* and *positive image of PE/PE teacher)* and three inhibitor themes (*perceived schedule constraints*, *contextually unrealistic program planning,* and *conflicting perceptions of PA and PE*) emerged across the full completer group and partial completer group. Participants expressed that support from multiple sources (administrator, classroom teachers, parents, and students) facilitated the initiation and implementation of their program by obtaining approval from administration and hands-on assistance from classroom teachers, parents, and students. They also expressed that having a positive image and being viewed as someone that was knowledgeable about PA facilitated respect and support from others in the school (administration, classroom teachers, and students). The inhibitor theme of perceived schedule constraints differed across completer groups. Partial completers mostly reported this inhibitor due to academically prioritized schedules, whereas full completers expressed schedule constraints due to playing multiple roles in their school. Contextually unrealistic program planning inhibited implementation for both groups due to unforeseen events such as bad weather or planning an initiative without considering the absence of contextual factors (e.g., lack of facilities/equipment). Participants also expressed conflicting perceptions of PA and PE as some PE teachers did not perceive PA as their responsibility. Table 3 presents the subthemes and representative data clips for each theme/subtheme.

## 5 Discussion

The purpose of this process evaluation was to determine the extent to which the CSPAP professional development program was delivered and implemented during its three-year lifespan. Six process aspects of the CSPAP professional development program were measured using the collection of mixed-method data. A summary of the main implications, encountered challenges and potential remedies to optimize future CSPAP professional learning opportunities are provided.

### 5.1 Recruitment and reach

The many, multilevel (national professional organization; state agencies, annual state conference, university PE teacher education programs) recruitment strategies employed for the professional development program resulted in relatively few training cohorts and interested teachers. To no surprise, incentives in the form of a travel stipend, free registration, and continuing education credits helped ensure 94% of the registered teachers attended a workshop they reportedly regarded highly. The popularity and utility of workshop incentives for increasing attendance rates is supported by previous teacher research (Forster et al., 2015, Qian et al., 2018). Furthermore, recruitment strategies used in the present evaluation did invoke some participation from secondary public-school teachers, a population seldom evaluated with regard to professional development (Whitworth and Chiu, 2017). Most recruited participants and full completers of the program were female, reiterating that female educators tend to be more interested in professional learning opportunities than their male counterparts (Czerniawski et al., 2016). There is a need for creative ways to recruit and reach male and secondary teachers for professional development activities, such as using males to recruit other males or tailoring training for males and females to address gender differences in learning (Cunningham and Watson, 2002, Li, 2016). It should also be noted that the geographic locations of the one-day workshops could have limited the ability to reach PE teachers from nearby states resulting in higher representation from specific states.

### 5.2 Dose delivered and received

The delivery of a highly-regarded workshop did not automatically entice teachers to participate in the follow-up process and receive their certification, as less than half of teachers remained involved and initiated the next criterion of submitting an artifact. Interview data revealed that removed cost to participate in and have access to all resources in the 12-month follow-up period facilitated participation in the workshop. Likewise, research has indicated that monetary rewards could be a good initial incentive, but not necessarily an enhancement, for teacher learning (Kyndt et al., 2016). Therefore, participants may have perceived subsequent costs to outweigh their progression through the professional development process beyond the initial one-day workshop. This potential limitation to continued involvement in the post-workshop criteria reinforces the importance of prolonged professional development. Research suggests that professional development over a longer duration is more successful for maintaining participation and changing teacher practices (Whitworth and Chiu, 2017). Nevertheless, once any post-follow-up barriers were overcome, a large percentage (79%) of teachers who submitted implementation plans in the current study remained involved until the end of the professional development program and received certification. Future research should investigate promising strategies for increasing planning interests and involvement besides reducing or eliminating follow-up costs (Moore et al., 2017). For instance, this study highlights the possibilities and benefits of networking and ensuring mentors are readily available, which other research has corroborated (Luft et al., 2011).

### 5.3 Fidelity and context

The current evaluation yielded some evidence suggesting that teachers may have chosen initiatives they perceived to be the easiest to implement. The most frequent CSPAP component targeted (during school) consisted of PA initiatives teachers expected to implement in the shortest length of time (4.2 months). The likely reoccurrence of the planned initiatives also varied greatly (e.g., brain breaks vs. family night). Initiatives were largely planned as a 5-step implementation process that included obtaining approval and buy-in from colleagues, especially from a school administrator. The common planning sequence included first obtaining administrative approval followed by preparation and implementation, which mirrors the initial steps in the CSPAP guide and emphasizes that administrator support is necessary for successful program launch and uptake (CDC, 2015). Also, among the different types and sources of perceived social support that exist (Wills and Shinar, 2000), emotional support (i.e., expression of encouragement), instrumental support (i.e., practical assistance), and validation support (i.e., others confirming one’s thoughts) appear to be highly regarded by CSPAP-trained teachers, particularly when it comes from administrators, classroom teachers, and community members. These forms of perceived social support were also reflected in the elevated image of PE and PE teacher facilitator.

An interesting finding was the fact that the evaluation of the implemented initiative was irregularly included in teachers’ submitted implementation plans (only appearing in 27% of them, mostly as step five), even though research substantiates the importance of data-driven decision making in education (Hamilton et al., 2020, Marsh et al., 2006). This discovery underscores teachers’ tendency to rely on personal experience rather than outside data sources for evaluation of their initiatives (Ingram et al., 2004). Multiple data sources, including varying stakeholder perspectives, can inform future work aiming to identify and facilitate a more diverse, interdisciplinary focus of the initiative beyond PA from the sole perspective of the PE teacher (Bowen, 2009, Kaittani et al., 2017). Completer group differences in reported inhibitors of implementation also highlight a helpful approach for overcoming perceived contextual barriers in the future. For example, partial completers tended to cite the academically-prioritized focus of their school schedule as a barrier rather than finding a way to adapt and utilize this focus to advance physical health (e.g., by integrating movement with academic activities; Russ et al., 2017). Generally, the CSPAP leader role attracts highly involved teachers who have hopeful (maybe idealistic) visions for their school, and this may be exacerbated among early adopters (Centeio et al., 2014, Glowacki et al., 2016). In this study, early adopters were the full completers who planned to implement initiatives with unavoidable challenges (e.g., weather, transportation, inadequate equipment or space available) and fulfilled several roles during implementation. This finding reaffirms that invested teachers are the best candidates to target for championing the comprehensive, multicomponent aspects of CSPAP (Centeio et al., 2014).

A common inhibitor to program implementation was the perceived added workload, especially among the partial completers who noted extra workload as the reason for not participating in follow-up. It can be argued that PE is experiencing an “identity crisis” (Deslatte and Carson, 2014), wherein some PE teachers see their job as “in the gym” only and view schoolwide PA as outside their responsibility. There may be a real fear that PE is being forgotten or further pushed to the periphery with the advancement of CSPAP as the national guiding model for increasing PE and physical activity opportunities in schools (CDC, 2017a). One helpful suggestion is for CSPAP advocates to consistently depict PE as the cornerstone in the messaging of CSPAP. Perhaps, the CSPEP framework (Webster et al., 2016), the proposed illustrative supplement of CSPAP (Webster et al., in press), or CSPAP research-to-practice handbook (Carson and Webster, 2020) may help garner unfettered buy-in among PE professionals. Overall, the facilitators and inhibitors identified in this evaluation are important considerations if the full potential of CSPAP professional development programs (i.e., PAL), and ultimately sustainable CSPAP implementation, are to be achieved.

## 6 Conclusions & implications

Despite not all CSPAP professional development criteria being completed by every participant, this mixed-method process evaluation reveals the unique perspectives of PE teachers with regard to schoolwide PA promotion and informs future efforts aiming to target and effectively support CSPAP leaders. Due to varying levels of teacher interest in and commitment to CSPAP training disclosed by the present evaluation, future work in this area should consider a tiered approach to CSPAP professional development, such as offering multilevel opportunities (e.g., workshops, longer courses) that address teachers’ perceived barriers to and facilitators of participation and implementation (Dauenhauer et al., 2018). Additionally, findings from this evaluation yield key public health implications by suggesting CSPAP can help portray an elevated image of PE that can permeate throughout the school and local community. As one district coordinator noted regarding the power of a CSPAP leader:

“Other teachers look at them as the leader…And they go to them and ask them for tips and classroom teachers will come to them and be like, ‘Oh, do you have any ideas of how I can teach yoga in my class?’ or ‘Do you mind leading a stretch break at our next staff meeting?’ So they’ve gotten outside of the gymnasium, which I think is what this is all about. Really—it allows them to feel empowered and you can really see a difference in how they come and teach every day, which is great.”

## Declaration of Competing Interest

The authors declare that they have no known competing financial interests or personal relationships that could have appeared to influence the work reported in this paper.

## Footnotes

1 The DPA certification program was transformed into the Physical Activity Leader (PAL) learning system in 2013 (SHAPE America, 2015b)

2 The National Association for Sport and Physical Education (NASPE), one of the five national associations of the American Alliance for Health, Physical Education, Recreation and Dance (AAHPERD), was the initial managing organization of the CSPAP professional development program known as the Director of Physical Activity (DPA) certification program. NASPE dissolved and unified under the AAHPERD name in 2013, and in 2014, was renamed to SHAPE America – Society of Health and Physical Educators.
